# Histone H3 Lysine 36 Methyltransferase Whsc1 Promotes the Association of Runx2 and p300 in the Activation of Bone-Related Genes

**DOI:** 10.1371/journal.pone.0106661

**Published:** 2014-09-04

**Authors:** Yu Fei Lee, Keisuke Nimura, Wan Ning Lo, Kotaro Saga, Yasufumi Kaneda

**Affiliations:** Division of Gene Therapy Science, Osaka University Graduate School of Medicine, Osaka, Japan; Texas A&M University, United States of America

## Abstract

The orchestration of histone modifiers is required to establish the epigenomic status that regulates gene expression during development. Whsc1 (Wolf-Hirschhorn Syndrome candidate 1), a histone H3 lysine 36 (H3K36) trimethyltransferase, is one of the major genes associated with Wolf-Hirshhorn syndrome, which is characterized by skeletal abnormalities. However, the role of Whsc1 in skeletal development remains unclear. Here, we show that Whsc1 regulates gene expression through Runt-related transcription factor (Runx) 2, a transcription factor central to bone development, and p300, a histone acetyltransferase, to promote bone differentiation. *Whsc1* ^−*/*−^ embryos exhibited defects in ossification in the occipital bone and sternum. Whsc1 knockdown in pre-osteoblast cells perturbed histone modification patterns in bone-related genes and led to defects in bone differentiation. Whsc1 increased the association of p300 with Runx2, activating the bone-related genes *Osteopontin* (*Opn*) and *Collagen type Ia* (*Col1a1*), and Whsc1 suppressed the overactivation of these genes *via* H3K36 trimethylation. Our results suggest that Whsc1 fine-tunes the expression of bone-related genes by acting as a modulator in balancing H3K36 trimethylation and histone acetylation. Our results provide novel insight into the mechanisms by which this histone methyltransferase regulates gene expression.

## Introduction

Histone modifications are crucial in ensuring proper embryonic development [1] [2]. For example, Wolf-Hirschhorn syndrome (WHS) is a syndrome closely associated with epigenetics. WHS is defined by craniofacial dysgenesis, growth delay, mental retardation, and heart malformations, among other characteristics [3] [4], and is caused by sub-telomeric deletions on the short arm of chromosome 4p16.3. *Whsc1* (WSH candidate 1, also known as NSD2 or MMSET), the gene that encodes the histone 3 lysine 36 (H3K36) trimethyltransferase Whsc1 [5], is deleted in every case of WHS [3], and this deletion is necessary for the occurrence of WHS [6]. Because WHS patients show skeletal abnormalities, such as sternal hypo-ossification [7], delayed growth, and craniofacial abnormalities [3] [8], it has been concluded that Whsc1 is involved in bone development. However, its function in skeletal development remains unknown.

Runt-related transcription factor (Runx) 2 plays a critical role during the differentiation of mesenchymal cells into osteoblasts [9]. Runx2 belongs to the Runx family of genes, which contain the conserved Runt DNA binding domain and are key regulators during development. Gene knockout models have helped elucidate the key functions of the Runx genes; Runx1 is associated with hematopoiesis, Runx2 with bone formation, and Runx3 with cytotoxic T-cell development [10]. Runx genes have many other roles in addition to their major functions and can be co-expressed in the same tissue. Although Runx1 plays a key role in hematopoiesis, it also cooperates with Runx2 [11] [12] in sternum development and in the early stages of mesenchymal differentiation [13]. Runx genes function as DNA binding transcription factors and interact with co-repressors or co-activators in the suppression or activation of genes. Runx2 represses the osteocalcin gene [14] when it interacts with the co-repressor histone deacetylase (HDAC) 3. In contrast, Runx2 activates matrix metalloproteinase-13 gene [15] when bound to p300, a co-activator histone acetyltransferase (HAT). The co-regulation of histone modification-related enzymes and bone-related transcription factors, including Runx2, has been found to be crucial in the regulation of bone cell differentiation genes [16].

Whsc1 contains the SET (suppressor of variegation, enhancer of zest and trithorax) domain, which specifically catalyzes histone 3 lysine 36 methylation (H3K36me). H3K36me can exist in non-methylated (me0) or mono-, di-, or tri- methylated forms (me1, me2, or me3, respectively). With respect to the methylation status of H3K36, H3K36me3 has been highly correlated with transcribed genomic regions from transcription start sites to transcription termination sites [17] [18]. Other HMTs that catalyze H3K36me3 include HYPB/SETD2 and NSD1 [19] [20] [21]. In yeast, SET2, the ortholog of HYPB/SETD2, methylates H3K36 in transcribed regions. Through its interaction with the elongating RNA polymerase II, cryptic transcription, in which transcription begins in the middle of the coding region, is inhibited via the suppression of histone turnover [22] [23]. HYPB/SETD2 acts in conjunction with RNA polymerase II, whereas Whsc1 interacts with transcription factors to prevent the overactivation of genes during heart and embryonic stem cell development [5].

In the present study, we observed that Whsc1 associates with Runx2 in the regulation of bone-related genes, ensuring proper skeletal development. In particular, Whsc1 fine-tunes the expression of bone-related genes through H3K36me3 and histone acetylation by promoting the interaction between Runx2 and p300. These findings suggest an essential role for Whsc1 with Runx2 and p300 in the activation of bone-related gene expression during bone development.

## Materials and Methods

### Ethics

We used mice in accordance with protocols approved by the Ethics Committee for Animal Experiments of the Osaka University Graduate School of Medicine.

### Cell culture and mice

Murine C3H 10T1/2 mesenchymal cells [24] and MC3T3-E1 murine pre-osteoblasts [25] were used in the *in* *vitro* studies. C3H 10T1/2 mesenchymal cells were grown in Dulbecco’s modified Eagle’s medium (DMEM) (Nacalai Tesque) supplemented with 10% fetal bovine serum (FBS), 100 U/ml penicillin, and 0.1 mg/ml streptomycin. MC3T3-E1 cells were cultured in α-modified Eagle’s medium (α-MEM) (GIBCO Catalogue: 12571-063) supplemented with 10% FBS, 100 U/ml penicillin, and 0.1 mg/ml streptomycin. Osteoblast differentiation was induced by the addition of 300 ng/ml bone morphogenetic protein (BMP)-2 (PEPROTECH) to the culture medium.

*Whsc1* ^−/−^ embryos were obtained from *Whsc1^+/^* ^−^ mice that had been backcrossed to C57BL/6 over 10 times. The targeting strategy was conducted as described in [5].

### Skeletal preparations

Embryos were fixed in 20% formalin for 2 days at room temperature and subsequently eviscerated. After dehydration in an increasing ethanol series up to 100% ethanol for 30 minutes each, the embryos were bleached with Dent’s fixative (5∶36∶9 ratio of aqueous H_2_O_2_:MeOH:DMSO, respectively) overnight. The embryos were then washed with a decreasing ethanol series, down to 70% ethanol, for 30 minutes each. Subsequently, the embryos were stained with 0.1% Alizarin red at 37°C overnight. The embryos were again dehydrated in an ethanol series, up to 100% ethanol, for 30 minutes each, followed by staining with Alcian blue solution for 2 days at 37°C. The embryos were then washed and placed in xylene for 2 hours to remove fats. Then, the embryos were cleared with a trypsin solution (30 ml:70 ml:1 g ratio of sodium tetraborate saturated solution:distilled water:trypsin, respectively) at 37°C and kept in 80% glycerol.

Micro CT imaging of newborn was obtained by in vivo micro X-ray CT system R_mCT2 (Rigaku).

### Alkaline phosphatase assay

MC3T3-E1 cells plated in 24-well plates were washed twice with Tris-buffered saline (TBS), scraped into 0.3 ml of 0.5% NP-40 containing 1 mM MgCl_2_ and 10 mM Tris (pH 7.5), and homogenized three times for 10 seconds each with a multi-bead shocker (Yasui Kikai). Then, the cell lysates were centrifuged for 5 minutes at 2000 rpm, and the supernatants were used for the enzyme assay. Alkaline phosphatase activity was assayed using a commercial kit (Wako Pure Chemicals Industries) with ρ-nitrophenylphosphate as a substrate. The enzymatic activity was expressed in units/µL defined by the release of 1 nmol of ρ-nitrophenylphosphate per minute at pH 9.8 and 37°C. This enzymatic activity was then normalized to the protein content of the sample. The protein content was determined using a bicinchoninic acid (BCA) protein assay kit (Thermo Scientific) with bovine serum albumin (BSA) as the standard.

### Plasmid construction

To create Runx1 and Runx2 plasmids for the overexpression experiments, mouse Runx1 and Runx2 were first amplified using polymerase chain reaction (PCR) from cDNA templates, which were reversed transcribed from the total mRNA of C3H 10T1/2 cells. Runx1, Runx2, and Runx2 deletion mutants were introduced into the pCAGIP-myc and pCAGIP-HA expression vectors using Gateway Technology (Invitrogen). The Whsc1 expression constructs have been previously described [5]. For the luciferase promoter assays, the *Osteopontin* (*Opn*) promoter (−1010 to +146) and the *Collagen type Ia* (*Col1a1*)-luciferase construct (−2320 to +183) were generated by PCR using mouse genomic DNA, and the resulting fragments were cloned into the pGL3-basic vector (Promega). The primer sequences are shown in Table S1.

### Transient plasmid transfections and targeted gene knockdown

FuGENE HD (Promega) was used to transfect plasmids into CH3 10T1/2 cells according to the manufacturer’s instructions. A mixture of 35 µl of FuGENE HD and 10 µg of DNA was used to transfect the cells seeded in a 10-cm dish. The Neon (Invitrogen) electroporation method was used to transfect plasmids and small interfering RNAs (siRNAs) at a final concentration of 100 µM into MC3T3-E1 cells. The following conditions were used for Neon: pulse voltage, 1600 V; pulse width, 10 ms; and pulse number, 3.

The siRNA method was used to knockdown the expression of targeted genes in MC3T3-E1 cells. Runx1, Runx2, Whsc1, or control siRNA (Sigma-Aldrich) were transfected into MC3T3-E1 cells using the Neon Transfection System (Invitrogen) as mentioned above. MC3T3-E1 cells were cultured for 1–2 days in a 12-well plate at a cell density of 1×10^5^ cells/well. The following siRNAs were obtained from MISSION (Sigma-Aldrich): siRunx1.6068 (SASI_Mm02_00306068), siRunx1.6069 (SASI_Mm02_00306069), siRunx1.6070 (SASI_Mm02_00306070), siRunx2.4517 (SASI_Mm01_00044517), siRunx2.4518 (SASI_Mm_00044518), siWhsc1.5204 (SASI_Mm02_00295204), and siWhsc1.5207 (SASI_Mm02_00295207).

### Cell fractionation

To obtain nuclear extracts for analyses, MC3T3-E1 (1×10^7^ cells/sample) and C3H 10T1/2 (3.7×10^6^ cells/sample) cells were extracted in 500 µl of nuclear isolation buffer (NIB) containing 0.15% NP-40 and EDTA-free ‘complete’ protease inhibitor cocktail (Roche) as previously described [26]. Cytoplasmic soluble proteins were separated from the nuclei by centrifugation at 15,000 rpm for 5 minutes at 4°C. Nuclear pellets were further treated with 1200 U of micrococcal nuclease (TaKaRa) in 250 µl of nuclear isolation buffer containing 200 mM NaCl at 25°C for 15 minutes. After incubation on ice for 10 minutes, 10 mM EDTA was added, and the samples were incubated on ice for 10 minutes. The nuclear extracts were separated from the pellets by centrifugation at 15,000 rpm for 5 minutes.

### Co-immunoprecipitation

C3H 10T1/2 cells were used to study the interactions between endogenous p300 and the overexpressed Runx and Whsc1 proteins. Nuclear extracts were isolated from C3H 10T1/2 cells 24 hours after transfection. Nuclear extracts were first pre-cleared with rabbit IgG AC (Santa Cruz Biotechnology) and then incubated overnight with HA-tagged beads to bind the Runx-HA-tagged proteins. The beads were washed 4 times using nuclear isolation buffer (NIB) containing 200 mM NaCl.

### RNA extraction and real-time PCR

To analyze gene expression, total RNA from embryonic stage 18.5 (E18.5) mouse bone tissues and MC3T3-E1 cells were first extracted using Isogen (Nippon Gene). For bone tissues, homogenization was performed before extraction. Tissues were homogenized three times at 2000 rpm for 10 seconds in 1 ml of Isogen and then centrifuged at 15,000 rpm for 5 minutes to remove cell debris. The supernatant was collected, and RNA extraction was performed according to the manufacturer’s instructions. In each experiment, an equal quantity of total RNA was reversed-transcribed into cDNA using Superscript III (Invitrogen). Quantitative PCR was performed using SYBR Premix Ex Taq (TaKaRa) and Real-time PCR Master Mix (TOYOBO). Amplifications were performed in a C1000 thermal cycler (Bio-Rad). The quantified mRNA levels were normalized to the expression of *Rplp2* (bone tissues) and *18S rRNA* (MC3T3-E1) mRNA (as an internal control). TaqMan probes and primer pairs specific for *Osteopontin* (*Spp1*) (TaqMan Mm00436767_m1), *Osteocalcin* (*Bglap1*) (TaqMan Mm03413826_mH), *Alkaline phosphatase* (Mm00475381_m1), *Collagen type Ia* (TaqMan Mm00801666_g1), *Runx1* (TaqMan Mm01213405_m1), and *Whsc1* (TaqMan Mm01211104_m1) were purchased from Applied Biosystems (Foster City, CA). The primers for *Runx2*, *Col10a1*, *Rplp2* (*Ribosomal protein, large P2*), and *18S* (*18S rRNA*) are shown in Table S1. Assays were performed in triplicate within a single experiment, and the average values were used for further analysis.

### Pre-osteoblast cell count

MC3T3-E1 cells were counted at 36 hours after transfection using a cell counter (Beckman Coulter Z) or a TC-10 Automated Cell Counter (Bio-Rad Laboratories).

### Luciferase reporter assay

The *Opn* or *Col1a1* luciferase reporter constructs and the expression constructs were co-transfected into C3H 10T1/2 cells that were seeded at a density of 2×10^4^ cells/well in 24-well plates one day prior to transfection. The ratios of the expression constructs were as follows: for the luciferase reporter construct, the *Renilla* plasmid ratio, as an internal control, was 5∶4∶1, and 0.5 µg of total DNA was transfected per well. The transfection reagent/DNA complex for 10 wells was made from 5 µg of DNA and 17.5 µl of FuGENE HD (Roche) in 250 µl of OPTI-MEM. A total of 25 µl of the resulting complex was added to each well. C646, a p300 inhibitor (Sigma-Aldrich), was dissolved in DMSO and was added to the appropriated groups 12 hours after transfection at a concentration of 45 nM. After 24 hours of incubation, luciferase activity was measured using the Dual Luciferase Reporter Assay system (Promega).

### Western blot analysis

Trypsinized MC3T3-E1 cells were washed once with PBS and dissolved in 2X sample buffer (1×10^5^ cells/10 µl). To denature the proteins, the samples were vortexed for 5 minutes and incubated at 100°C for 5 minutes, and this process was repeated 3 times. Immunoprecipitation samples were also analyzed by western blot analysis. Protein samples (20 µl per lane) were separated by SDS-PAGE and transferred onto a Hybond-P PVDF membrane (GE Healthcare). The following antibodies were used for western blot analysis: anti-histone H3 (1∶1000, ab1791, Abcam), anti-Runx1 (1∶500, 8529, Cell Signaling Technology), anti-Runx2 (1∶1000, 8486, Cell Signaling Technology), anti-p300 (1∶100, sc-585, Santa Cruz Biotechnology), and anti-Whsc1 (1∶200) [5]. All of the primary antibodies were detected using secondary antibodies conjugated to horseradish peroxidase (anti-rabbit IgG, GE healthcare). A ReliaBLOT (Bethyl Laboratories) kit was used to reduce the IgG heavy chains in the IP samples. To strip the blots, the membranes were incubated with WB Stripping Solution Strong (Nacalai Tesque) for 15 minutes at room temperature.

### Chromatin immunoprecipitation (ChIP) qPCR

MC3T3-E1 cells were transfected with control siRNA or siRNA directed against Whsc1 and were cultured in BMP-2 for 6 days. Nuclei was isolated by NIB containing NP40 from 0.5×10^7^ cells, then chromatin was digested at 25°C for 30 min by 4.8 U ml^−1^ micrococcal nuclease (TaKaRa) in 250 µl of NIB contained 400 mM NaCl as described previously [26]. The nuclear extract was diluted to 200 mM NaCl by 250 µl of NIB contained 0 mM NaCl. Chromatin was immunoprecipitaed with anti-Runx2 (2.5 µg, 8486, Cell Signaling Technology), anti-H3K36me3 (5 µg, ab9050, abcam), and anti-H4ac (5 µl, 06-866, Millipore) antibodies, then purified using a QIAquick PCR purification kit (Qiagen) [27]. THUNDERBIRD™ SYBR® qPCR mix (TOYOBO) was used for real-time PCR analysis. The primer sequences are shown in Table S1.

### Statistical analysis

Statistical analyses were performed using JMP 9 software (SAS Institute). Data were compared using the two-tailed Student’s *t*-test for pairwise comparisons against control and the Tukey-Kramer HSD test for multiple comparisons. Significance was defined as *P*<0.05.

## Results

### Skeletal abnormalities in the Whsc1 knockout mice

**Figure 1:**
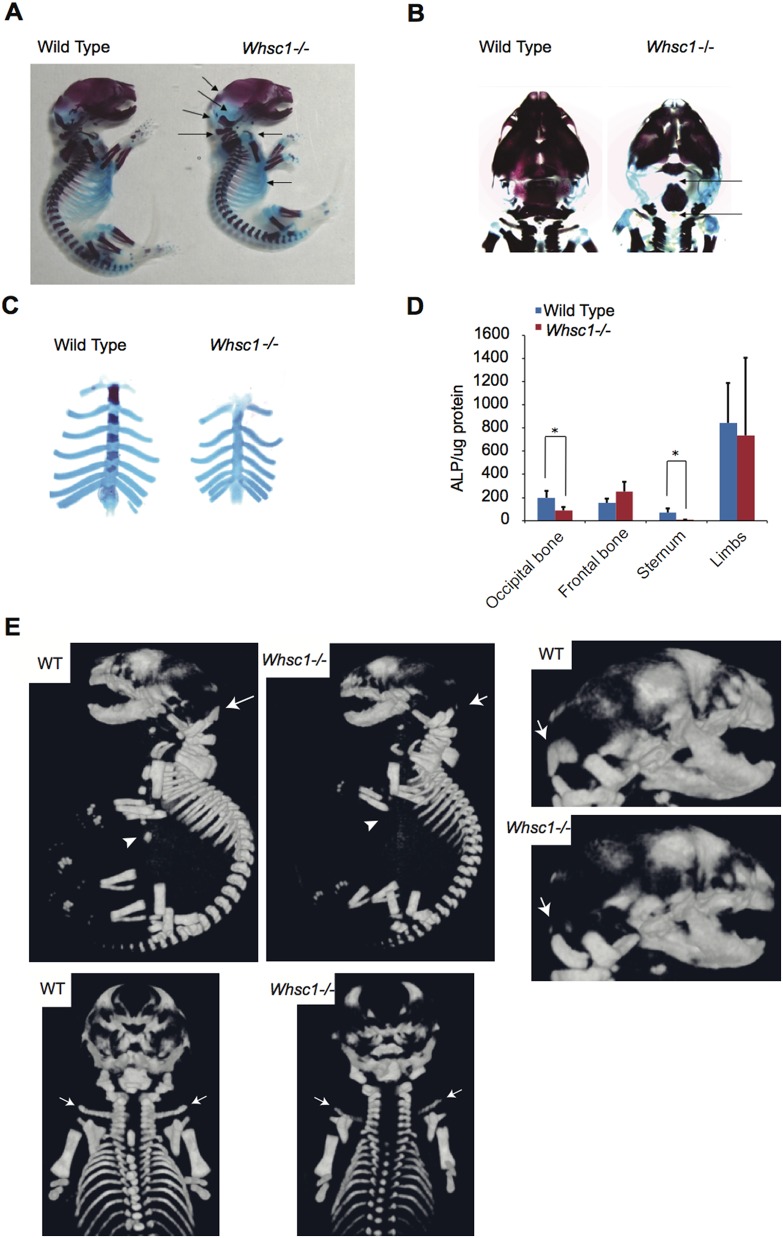
Skeletal abnormalities in E18.5 *Whsc1* ^−*/*−^ embryos. (A–C) Calcified tissues from wild type and *Whsc1* ^−*/*−^ embryos were stained red with Alizarin red, and the cartilage was stained blue with Alcian blue. (A) Whole skeletal structure. Arrows represent the areas of the skeletal structure lacking ossification. (B) Top view of the cranial bones. Arrows denote the extensive lack of ossification in the occipital bone. (C) Ribs with sternum. (D) Alkaline phosphatase assay on bone samples from wild type and *Whsc1* ^−*/*−^ embryos. *, *P*<0.05. (E) Micro-CT imaging of mineralized bones in postnatal 0 day wild type and *Whsc1* ^−*/*−^ newborn. Arrow heads indicate sternum. Arrows indicate occipital bone or clavicles.

To study the aberrations of the skeletal system, whole skeletal structures of E18.5 wild type and *Whsc1* ^−*/*−^ embryos were fixed and stained with Alizarin red to stain the calcified bones and Alcian blue to stain the cartilage. *Whsc1* ^−*/*−^ embryos had significant disruptions of ossification in cranial bone elements, including the occipital bone and periotic bone, as denoted by arrows in Fig. 1A. An enlarged top view of the cranial bone indicated a severe lack of ossification in the occipital bone (Fig. 1B). In addition to the abnormal cranial bone ossification, the embryo had insufficient ossification in the sternum (Fig. 1A and 1C). We next examined the alkaline phosphatase (Alp) activity as an early marker for osteoblast differentiation. Whsc1 deficiency significantly decreased the Alp activity in the occipital bone and sternum (Fig. 1D). To examine the mineralization aberrations of *Whsc1* ^−*/*−^ bone, *Whsc1* ^−*/*−^ newborn was analyzed by micro X-ray CT system, because *Whsc1* knockout is lethal after birth [5]. *Whsc1* ^−*/*−^ newborn showed mineralization deficiency in occipital bone, sternum, and clavicles (Fig. 1E). These results suggest that Whsc1 is involved in the regulation of skeletal development, notably in the sternum and occipital bone.

### Whsc1 deficiency decreased the expression of bone development-related genes

**Figure 2:**
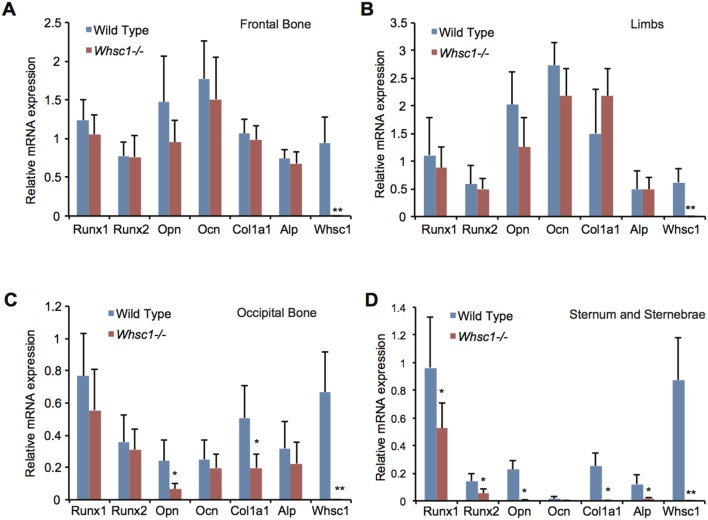
Whsc1 deficiency decreases the expression of bone tissue-related genes in E18.5 occipital bones, sternum and sternebrae. (A–D) Gene expression in *Whsc1* ^−*/*−^ E18.5 bone tissues was measured by qRT-PCR. Averages from six embryos of each genotype group are shown. Values were normalized to *Rplp2*. Error bars represent the s.d. *, *P*<0.05. **, *P*<0.001.

To understand the genetic basis of the ossification deficiencies observed in the occipital bone and sternum of *Whsc1* ^−*/*−^ embryos, we examined the expression levels of different genes in several bone tissues of E18.5 *Whsc1* ^−*/*−^ embryos. Whsc1 deficiency did not change the expression levels of the *Runx1* and *Runx2* genes, the critical transcription factors for bone development, or the bone-related proteins *Opn*, *Osteocalcin* (*Ocn)*, *Col1a1*, and *Alp* in the frontal bones (Fig. 2A) and limbs (Fig. 2B). In the occipital bone, Whsc1 deficiency decreased the expression of *Opn* and *Col1a1* but not *Runx1* and *Runx2* (Fig. 2C). The sternum of the *Whsc1* ^−*/*−^ embryos exhibited a drastic decrease in the expression of both transcription factors and bone-related proteins (Fig. 2D and Fig. S1). These results suggest that Whsc1 plays critical roles in bone differentiation in the sternum and occipital bone.

### Whsc1 is involved in osteoblast differentiation but not in proliferation

**Figure 3:**
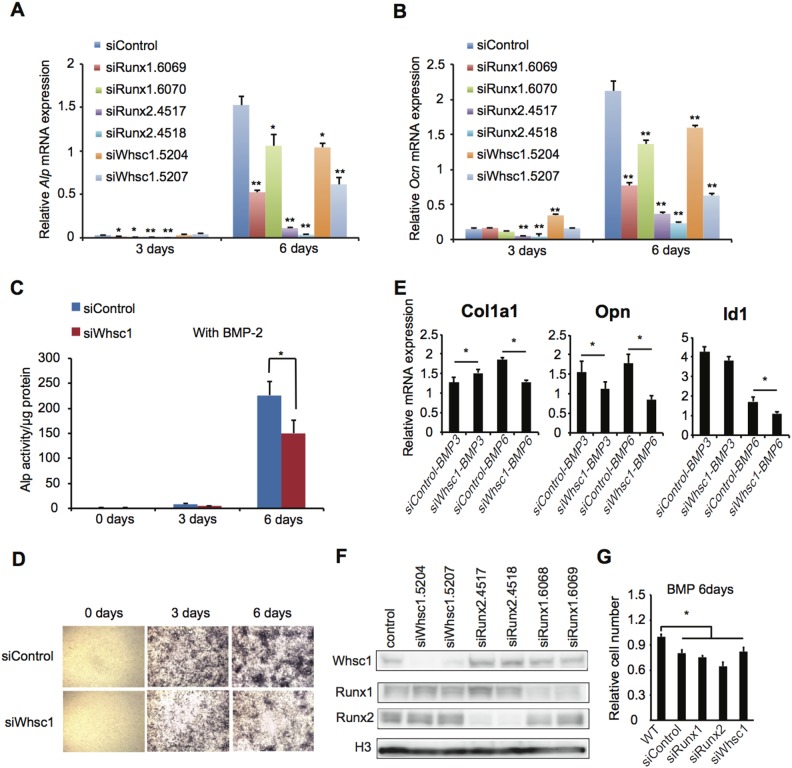
Whsc1 deficiency inhibits osteoblast differentiation but not cell proliferation. (A, B) *Alp* (A) and *Ocn* (B) mRNA expression during bone differentiation was measured by qRT-PCR. MC3T3-E1 cells were transfected with the indicated siRNAs and differentiated by incubation with BMP-2. Values were normalized to the expression of *18S*. (C, D) Alp activity was assessed using an alkaline phosphatase assay (C) or through Alp staining (D) in MC3T3-E1 cells transfected with the indicated siRNAs and in the presence or absence of BMP-2. (E) mRNA expression during bone differentiation was measured by qRT-PCR. MC3T3-E1 cells transfected with the indicated siRNAs were differentiated by 3 days (BMP3) and 6 days (BMP6) incubation with BMP-2. Values were normalized to the expression of *18S*. (F) MC3T3-E1 cells transfected with the indicated siRNAs were analyzed by western blot analysis with the indicated antibodies. (G) Cell proliferation of MC3T3-E1 cells transfected with the indicated siRNAs. MC3T3-E1 cells were differentiated by 6 days incubation with BMP-2. The value of wild type was set to 1.0. For all panels, Error bars represent the s.d. *, *P*<0.05. **, *P*<0.01.

To study the role of Whsc1 in bone differentiation, we repressed Whsc1 expression in pre-osteoblastic MC3T3-E1 cells. Bone differentiation was induced by the administration of BMP-2, which is known to induce osteoblast differentiation [28]. Cells cultured for 3 days showed a minimal increase in osteoblast differentiation-related gene expression compared with cells cultured for 6 days (Fig. 3A, 3B, and Fig. S2). Therefore, MC3T3-E1 cells started differentiating after 3 days in our method. Significant decreases in the levels of the differentiation-related markers *Alp* (Fig. 3A) and *Ocn* (Fig. 3B) were observed in the Whsc1-, Runx1-, and Runx2-depleted cells, indicating that these proteins are important for osteoblast differentiation. The decrease in gene expression was especially significant in the Runx2-knockdown cells, which is consistent with the report that Runx2 directly regulates osteoblast-specific genes, such as *Opn* [29]. Whsc1-knockdown cells exhibited a substantial difference in Alp activity after 6 days of BMP-2 treatment (Fig. 3C). Histochemical Alp staining further supports the above data, with lower Alp staining in the Whsc1-knockdown cells compared with the controls (Fig. 3D). Whsc1-knockdown also decreased *Col1a1*, *Opn*, and *Id1* expression in MC3T3-E1 cells after 6 days of BMP-2 treatment (Fig. 3E). Taken together, the significant decrease in osteoblast differentiation-related genes and the decrease in Alp production observed in Whsc1-knockdown cells indicate that Whsc1 is necessary for osteoblast differentiation at least in occipital bone and sternum.

Next, to exclude the possibility that Whsc1 affects the proliferation of pre-osteoblast cells, we examined whether Whsc1 knockdown decreases cell proliferation. Whsc1 knockdown did not decrease the expression of Runx1 or Runx2 in pre-osteoblast MC3T3-E1 cells, nor did depletion of Runx1 or Runx2 affect Whsc1 expression (Fig. 3F). No significant differences in the relative cell numbers were observed between the control and the Whsc1-, Runx1-, or Runx2-depleted cells after 6 days of BMP-2 treatment (Fig. 3G), indicating that the Whsc1 and Runx genes do not regulate cell proliferation before differentiation. Therefore, these results suggest that Whsc1, Runx1, and Runx2 are important for osteoblast differentiation but not for the proliferation of pre-osteoblasts.

### Whsc1 interacts with the skeletal transcription factor Runx2 and its co-activator p300

**Figure 4:**
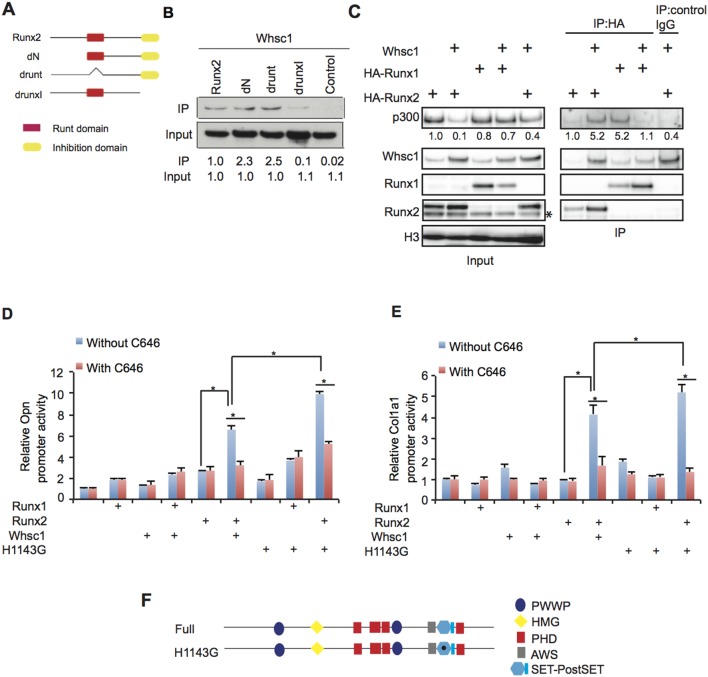
Whsc1 promotes the association between Runx2 and p300. (A) Schematic diagram of the mouse full-length and deletion Runx2 constructs. dN, deletion of the N-terminus; drunt, deletion of the Runt domain; drunxI, deletion of the Runx inhibition domain. (B) Western blot analysis showing the co-immunoprecipitation of Whsc1 by the full-length and deletion Runx2. (C) HA-tagged Runx1 and Runx2 were immunoprecipitated from C3H 10T1/2 cells transfected with the indicated expression vectors. The co-immunoprecipitation of Whsc1 and p300 and the input proteins were analyzed by western blot analysis. *Bottom bands represent the endogenous Runx2 expression in CH3 10T1/2 cells. (D, E) OPN (D) and Col1a1 (E) promoter activity in CH3 10T1/2 cells transfected with the indicated expression vectors. p300 activity was inhibited by C646. The data were normalized to the controls, and representative data from three independent experiments are shown. Error bars represent the s.d. (n = 4); *, *P*<0.001. (F) Schematic of mouse full-length Whsc1 and the Whsc1 mutant H1143G. PWWP, domain with the conserved PWWP motif; HMG, high-mobility group box; PHD, plant homeotic domain; AWS, associated with SET; SET-PostSET, Su (Var) 3–9, Enhancer-of-zeste, trithorax domain.

Next, we examined whether Whsc1 was associated with Runx2. Whsc1 co-immunoprecipitated with Runx2 as well as the dN and drunt truncated proteins, but not it did not co-immunoprecipitate with the drunxI truncated protein (Fig. 4A and 4B). This suggests that Runx2 interacts with Whsc1 through the runxI domain and hence Runx2 may associate with Whsc1 in the promotion of osteoblast differentiation. The histone acetyltransferase (HAT) p300 is known to interact with Runx1 [30] and Runx2 [31] during transcription activation. In heart development, *Whsc1* ^−*/*−^ led to a decrease in the H3K36me3 levels, and increased gene expression was observed [5]. However, the expression of bone-related genes was decreased in *Whsc1* ^−*/*−^ embryos (Fig. 2C and 2D). Thus, Whsc1 could recruit p300 to the Runx complex. Indeed, Whsc1 overexpression increased the association of p300 with Runx2 (Fig. 4C). Although interactions between p300, Runx1, and Whsc1 were detected, no increase was observed in the association between p300 and Runx1 (Fig. 4C). Non-specific binding of Whsc1 to control IgG was found, but p300 was not precipitated at the same time, suggesting that Whsc1 that was not involved in Runx2 complex was non-specifically precipitated with control IgG (Fig. 4C). Therefore, these results suggest that Whsc1 promotes the association of p300 and Runx2 but not the association of p300 and Runx1.

### Whsc1 is crucial for *Opn* and *Col1a1* gene activation

To reveal the function of the association of the Runx proteins with Whsc1 and p300 in promoting osteoblast differentiation, we examined whether these factors regulated *Opn* and *Col1a1* promoter activity. When Whsc1 and Runx2 were overexpressed, *Opn* and *Col1a1* promoter activity was significantly increased (Fig. 4D and 4E). In contrast, overexpression of Runx1 and Whsc1 did not increase *Opn* and *Col1a1* promoter activity (Fig. 4D and 4E). Furthermore, the p300 inhibitor C646 repressed the activation of the *Opn* and *Col1a1* promoters following Whsc1 and Runx2 overexpression (Fig. 4D and 4E). A mutation in the SET domain of Whsc1 (H1143G), which disabled the histone methyltransferase (HMT) ability of Whsc1 (Fig. 4F) [5], was also used to investigate the effect of H3K36me3 loss on promoter activation. Overexpression of H1143G and Runx2 caused stronger promoter activity compared with wild type Whsc1 and Runx2 overexpression (Fig. 4D and 4E) in a way that is consistent with the report that the H3K36me3 activity of Whsc1 represses overactivation of *Pdgfra* gene in heart development [5]. These data suggest that Whsc1, together with Runx2 and p300, is important for promoter activation, and its HMT activity modulates transcription level of the *Opn* and *Col1a1* genes.

### Whsc1 deficiency decreases Runx2 binding and histone H4 acetylation in target genes

**Figure 5:**
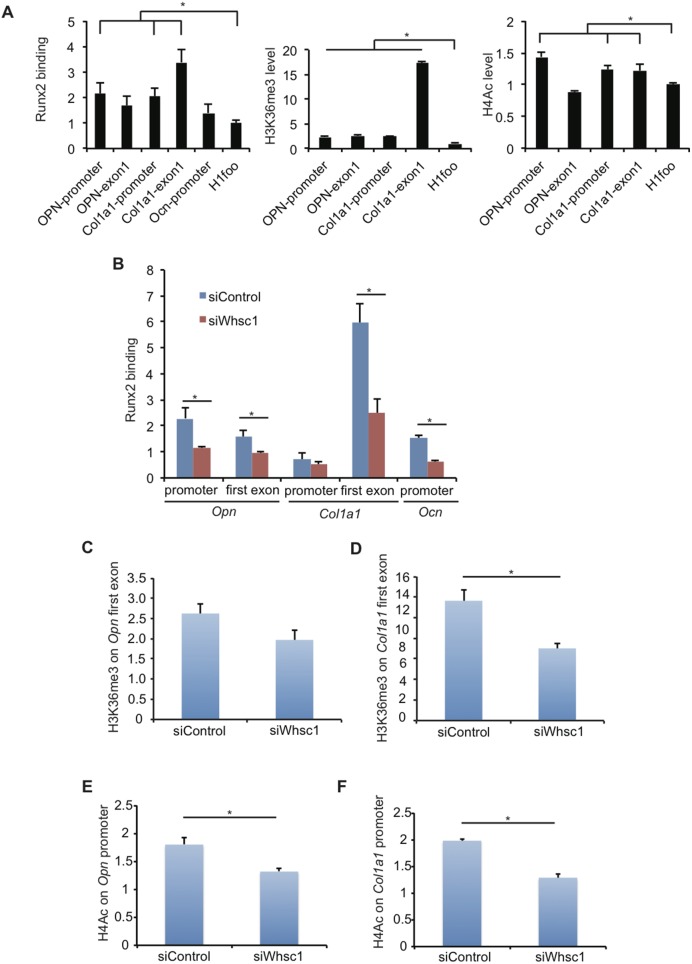
Whsc1 is required for Runx2 binding, H3K36me3, and H4 acetylation of the *Opn* and *Col1a1* genes. (A–F) Runx2, H3K36me3, and H4ac occupancy was examined by ChIP qPCR in MC3T3-E1 cells differentiated by 6 days incubation with BMP-2. (A) Runx2, H3K36me3, and H4ac occupancy in wild type MC3T3-E1 cells. Runx2 (B), H3K36me3 (C, D) and H4Ac (E, F) occupancy at the indicated regions of the *Opn* and *Col1a1* genes were examined in MC3T3-E1 cells transfected with the indicated siRNAs. The results are relative to 2% input and normalized to *H1foo*. Error bars indicate the s.d. (n = 3). *, *P*<0.05.

To confirm the effect of Whsc1 on the *Opn* and *Col1a1* genomic regions, Runx2 and the histone status at the *Opn* and *Col1a1* loci were examined using ChIP qPCR. First, We examined Runx2 binding to these genes in wild type MC3T3-E1 cells in 6 days culture with BMP2. Runx2 bound to *Opn* and *Col1a1* loci, compared with *Ocn* promoter including Runx2 binding sites [32] as positive control and *H1foo*, an oocyte specific gene, as negative control (Fig. 5A). H3K36me3 was highly enriched at *Col1a1* exon1 region and moderately enriched at other regions compared to *H1foo* (Fig. 5A). The acetylation of H4 (H4ac) was enriched at *Opn* promoter, *Col1a1* promoter, and *Col1a1* exon1 regions (Fig. 5A). Whsc1 knockdown significantly decreased Runx2 binding to the promoter and first exon regions of *Opn*, the first exon of *Col1a1*, and the promoter of *Ocn* genes in 6 days BMP-2-treated MC3T3-E1 cells (Fig. 5B). H3K36me3 levels in the first exon regions were also decreased in Whsc1-depleted MC3T3 cells (Fig. 5C and 5D). H4ac was measured to assess the effect of p300. H4Ac levels were decreased at the promoter of *Opn* and *Col1a1* in Whsc1-depleted cells (Fig. 5E and 5F). These results suggest that Whsc1 is important for recruiting Runx2 and p300 to the promoters of bone-related genes.

## Discussion

Our results provide evidence for the role of Whsc1 in skeletal development. We demonstrated that Whsc1, a H3K36 trimethyltransferase, is crucial in modulating the transcriptional activation of bone-related genes, such as *Opn* and *Col1a1*, through its associations with Runx2 and p300. In E18.5 *Whsc1* ^−*/*−^ embryos and newborns, significant disruptions in ossification were observed in the sternum (Fig. 1A, 1C, and 1E) and some cranial bone elements, particularly in the occipital bone (Fig. 1A, 1B, and 1E), but not in frontal bone and limb (Fig. 1A and 1E). Setd2 is also reported to have the activity of H3K36me3 [20] [33]. Because Whsc1 and Setd2 have the same enzymatic activity, they might have redundant function in the regulation of gene expression during bone development. Ossification disruption by Whsc1 deficiency in selective bones might be due to Setd2 rescuing Whsc1 deficiency in frontal bone and limb. The cranial bone defects observed in the *Whsc1* ^−*/*−^ embryos may explain the craniofacial defects observed in WHS patients. The phenotype of *Whsc1* ^−*/*−^ embryos affirms a link between Whsc1 and skeletal development. Whsc1 deficiency significantly decreased Alp activity (Fig. 1D) and *Opn* and *Col1a1* gene expression (Fig. 2C and 2D) in the sternum and occipital bone. Furthermore, Whsc1 knockdown in MC3T3-E1 cells also disrupted the progress of bone differentiation without suppressing cell proliferation (Fig. 3). However the gene expression of *Ocn*, a marker of mineralization, was not significantly changed in *Whsc1* ^−*/*−^ occipital bone and sternum (Fig. 2C and 2D), suggesting that Whsc1 deficiency may also affect early commitment or the numbers of osteoprogenitors. These results indicate that Whsc1 is essential to ensure proper ossification through the regulation of these bone-related genes.

Skeletal development is an intricate process that requires stringent control of gene activation and suppression by a variety of transcription factors [34]. Because Whsc1 interacts with heart transcription factors to ensure proper heart development [5], Whsc1 most likely associates with bone transcription factors. Therefore, because Runx transcription factors play pivotal roles in skeletal development, we focused on the association of Whsc1 with the Runx genes. Because the mRNA expression of Runx1 and Runx2 in the sternums of *Whsc1* ^−*/*−^ embryos (Fig. 1D) was decreased, we sought to determine whether Whsc1 directly regulated the Runx genes. Whsc1 knockdown did not substantially alter Runx protein expression (Fig. 3F). This result suggests the possibility that defects in bone differentiation caused by Whsc1 deficiency are not mediated by a decrease in Runx protein expression. Therefore, Whsc1 may instead act as a co-regulator of Runx proteins in a manner similar to the association between Whsc1 and Nkx2-5 observed during heart development [5].

Because Whsc1 may be associated with Runx in the promotion of osteoblast differentiation, we sought to confirm that this complex is involved in the transcriptional control of bone-related genes. We initially thought that Runx1 may be more closely associated with Whsc1 because conditional Runx1 knockout embryos [11] exhibited a similar lack of ossification in the sternum and occipital bone. However, our results indicated that although Runx1 was associated with Whsc1 (Fig. 4C), the combination of Runx1 with Whsc1 did not significantly enhance *Opn* and *Col1a1* promoter activity, unlike the significant enhancement observed for Runx2 and Whsc1 (Fig. 4D and 4E). Thus, our results indicate that Whsc1 regulates bone-related gene expression in conjunction with Runx2.

Although H3K36me3 is a mark of transcribed genomic regions and has the function to repress overactivation of transcription in active genes [5], we showed that Whsc1 is a positive regulator of bone-related genes (Fig. 4D and 4E). These data imply that Whsc1 requires co-activators for the activation of promoters. Histone acetyltransferases such as p300, are involved in transcription activation [35]. In addition, p300 recruitment to Runx2 has also been reported to play a role in the transcriptional activation of bone-related genes [31], suggesting that HATs, such as p300, may be associated with Whsc1 and Runx2. Our co-immunoprecipitation results indicated an association between Whsc1, Runx2, and p300 (Fig. 4C). Moreover, Whsc1 promoted the association between Runx2 and p300 (Fig. 4C). The requirement of p300 for the activation of *Opn* and *Col1a1* was further demonstrated by the decreased promoter activity after the addition of the p300 inhibitor C646 (Fig. 4D and 4E). In addition, the level of H4Ac, which is deposited by p300, was correlated with Whsc1 (Fig. 5D and 5E). Although H3K36me3 catalyzed by the HMT activity of Whsc1 was not required for recruiting Runx2 and p300 to promoter and for activating the bone differentiation genes, its loss led to the overactivation of the promoters (Fig. 4D and 4E). Thus, H3K36me3 catalyzed by Whsc1 allows for repressive mechanisms to prevent overactivation of target genes. Therefore, we propose that the *Opn* and *Col1a1* promoters are transcriptionally regulated during osteoblast differentiation. Whsc1 acts as a scaffold protein to recruit p300, thus promoting the association between Runx2 and p300. The activation of the *Opn* and *Col1a1* genes occurs in the presence of this activation complex, with H4Ac catalyzed by p300. Whsc1 also deposits H3K36me3 through its HMT activity on the first exon region (Fig. 5C and 5D) and this represses overactivation. Together with the activating acetylation marks of p300, the proper level of promoter activation occurs. Runx2 was reported to regulate bone differentiation in dose-dependent manner, suggesting that a critical threshold concentration of Runx2 is required for adequate transcription level [36] [37]. Thus, the function of Runx2 might be finely regulated for precise gene expression. Runx2-associating factors including Whsc1 and p300 might be critical for Runx2 activity to activate bone differentiation genes, because Whsc1 knockdown decrease not only Runx2 binding to the target regions of *Opn* and *Col1a1* genes but also histone acetylation on the promoter of these genes (Fig. 5). Furthermore, the embryos with 50% of the wild type Runx2 level show severe defect in occipital bone, compared with frontal bone [36], which is similar to *Whsc1* ^−*/*−^ embryos. These suggest that Whsc1 fine-tunes the transcription of the *Opn* and *Col1a1* genes with Runx2 and p300.

In conclusion, we propose a possible cross-talk pathway between Whsc1, Runx2, and p300 in the transcriptional activation of bone-related genes, leading to proper skeletal development at least in occipital bone and sternum.

## Supporting Information

Figure S1 **Col10a1expression in Whsc1** ^−**/**−^ **E18.5 bone tissues was measured by qRT-PCR.** Averages from six embryos of each genotype group are shown. Values were normalized to Rplp2. Error bars represent the s.d. *, P<0.05.
(TIFF)

Figure S2 **Effects of electroporation of siRNA to MC3T3-E1 cells.** Gene expression was examined by qRT-PCR and normalized to 18S. BMP3, 3 days culture with BMP2. BMP6, 6 days culture with BMP2. n = 3. Error bars indicate SD. *, p<0.05. N.S., not significant.
(TIFF)

Table S1 **Primers used in this research.**
(TIFF)
