# Supplementary figures and images for: Histone H3 Lysine 36 Methyltransferase Whsc1 Promotes the Association of Runx2 and p300 in the Activation of Bone-Related Genes
Source: PLoS One. 2014 Sep 4;9(9):e106661. doi: 10.1371/journal.pone.0106661 (PMC4154728; doi:10.1371/journal.pone.0106661)

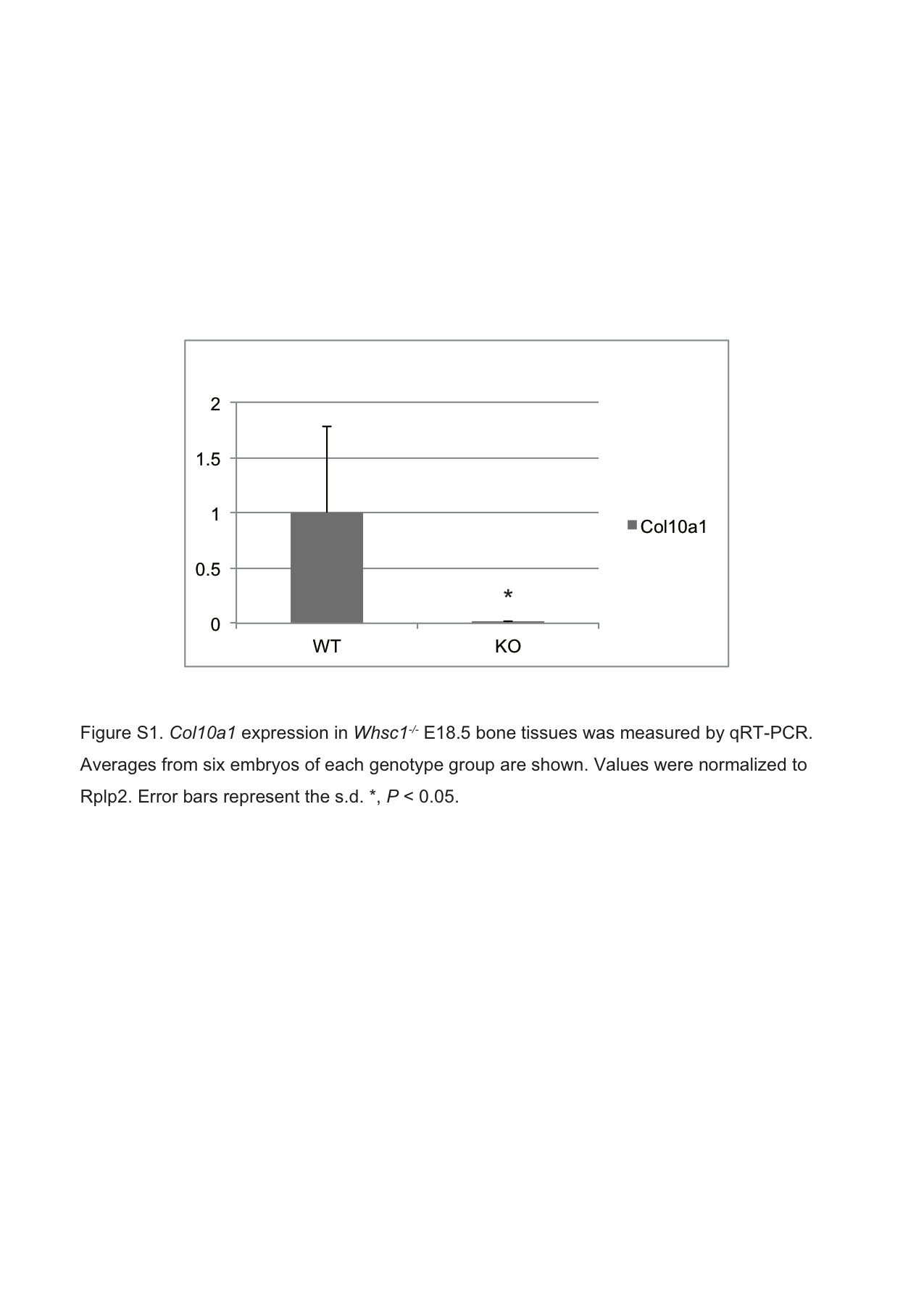

Supplement: Figure S1 — Col10a1expression in Whsc1 −/− E18.5 bone tissues was measured by qRT-PCR. Averages from six embryos of each genotype group are shown. Values were normalized to Rplp2. Error bars represent the s.d. *, P<0.05. (TIFF) [file pone.0106661.s001.tiff]

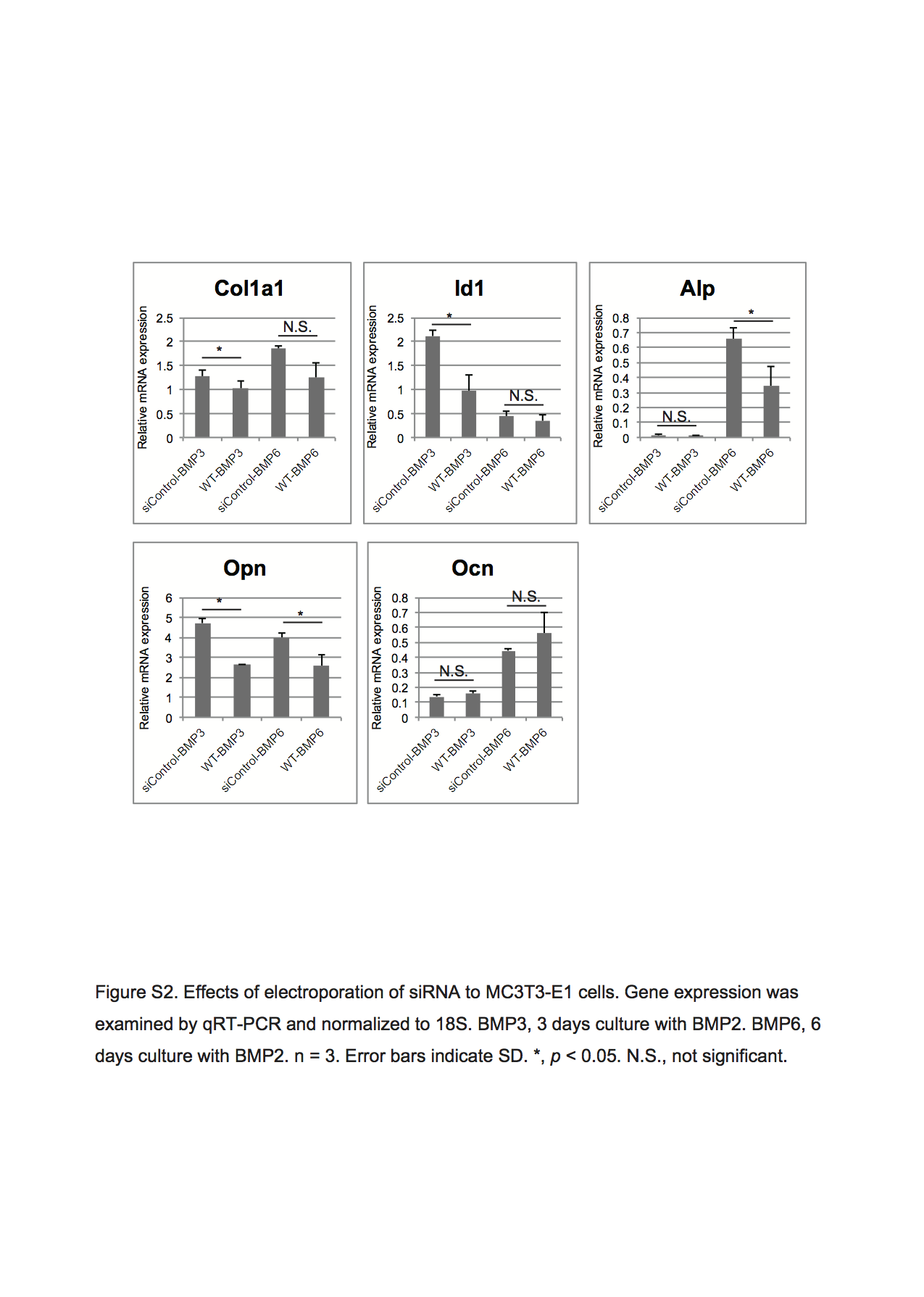

Supplement: Figure S2 — Effects of electroporation of siRNA to MC3T3-E1 cells. Gene expression was examined by qRT-PCR and normalized to 18S. BMP3, 3 days culture with BMP2. BMP6, 6 days culture with BMP2. n = 3. Error bars indicate SD. *, p<0.05. N.S., not significant. (TIFF) [file pone.0106661.s002.tiff]

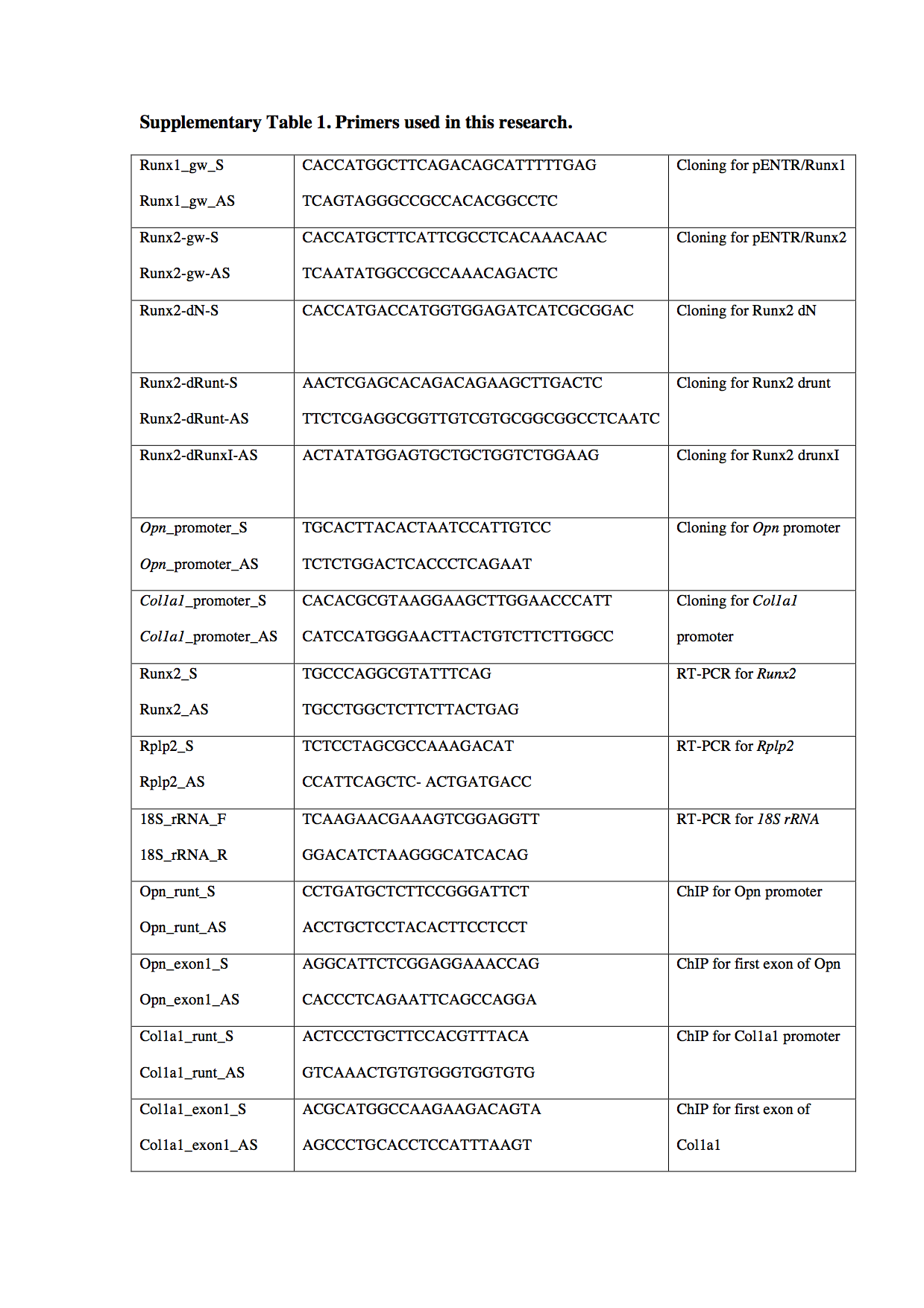

Supplement: Table S1 — Primers used in this research. (TIFF) [file pone.0106661.s003.tiff]
